# Integrated Genomic Analyses From Low-Depth Sequencing Help Resolve Phylogenetic Incongruence in the Bamboos (Poaceae: Bambusoideae)

**DOI:** 10.3389/fpls.2021.725728

**Published:** 2021-09-03

**Authors:** Domitille Chalopin, Lynn G. Clark, William P. Wysocki, Minkyu Park, Melvin R. Duvall, Jeffrey L. Bennetzen

**Affiliations:** ^1^Department of Genetics, University of Georgia, Athens, GA, United States; ^2^Department of Ecology, Evolution, and Organismal Biology, Iowa State University, Ames, IA, United States; ^3^Center for Translational Data Science, University of Chicago, Chicago, IL, United States; ^4^Department of Biology and Institute for the Study of the Environment, Sustainability, and Energy, Northern Illinois University, DeKalb, IL, United States

**Keywords:** Arundinarieae, Bambuseae, biased fractionation, diploidization, genome dominance, mobilome, Olyreae, plastome evolution

## Abstract

The bamboos (Bambusoideae, Poaceae) comprise a major grass lineage with a complex evolutionary history involving ancient hybridization and allopolyploidy. About 1700 described species are classified into three tribes, Olyreae (herbaceous bamboos), Bambuseae (tropical woody bamboos), and Arundinarieae (temperate woody bamboos). Nuclear analyses strongly support monophyly of the woody tribes, whereas plastome analyses strongly support paraphyly, with Bambuseae sister to Olyreae. Our objectives were to clarify the origin(s) of the woody bamboo tribes and resolve the nuclear vs. plastid conflict using genomic tools. For the first time, plastid and nuclear genomic information from the same bamboo species were combined in a single study. We sampled 51 species of bamboos representing the three tribes, estimated their genome sizes and generated low-depth sample sequence data, from which plastomes were assembled and nuclear repeats were analyzed. The distribution of repeat families was found to agree with nuclear gene phylogenies, but also provides novel insights into nuclear evolutionary history. We infer two early, independent hybridization events, one between an Olyreae ancestor and a woody ancestor giving rise to the two Bambuseae lineages, and another between two woody ancestors giving rise to the Arundinarieae. Retention of the Olyreae plastome associated with differential dominance of nuclear genomes and subsequent diploidization in some lineages explains the paraphyly observed in plastome phylogenetic estimations. We confirm ancient hybridization and allopolyploidy in the origins of the extant woody bamboo lineages and propose biased fractionation and diploidization as important factors in their evolution.

## Introduction

The bamboos (Poaceae: Bambusoideae), with ∼1700 species, are the third largest grass subfamily and represent the only major clade of grasses to diversify primarily in association with forests (Clark et al., 2015; Soreng et al., 2017; Clark and Oliveira, 2018). The bamboos are well supported as monophyletic and comprise three strongly supported lineages recognized as tribes: Arundinarieae (temperate woody bamboos, ∼580 species), Bambuseae (tropical woody bamboos, ∼1,000 species) and Olyreae (herbaceous bamboos, ∼124 species) (Kelchner and Bamboo Phylogeny Group, 2013; Clark et al., 2015; Wysocki et al., 2015; Zhang et al., 2016; Soreng et al., 2017; Ruiz-Sanchez et al., 2021). The woody bamboos (Arundinarieae and Bambuseae) share the woody syndrome, including gregarious monocarpy in most (Guerreiro, 2014; Clark et al., 2015; Wysocki et al., 2015), whereas the herbaceous bamboos (Olyreae) have relatively weakly lignified culms, restricted vegetative branching, and virtually all exhibit seasonal flowering (Clark et al., 2015).

**FIGURE 1:**
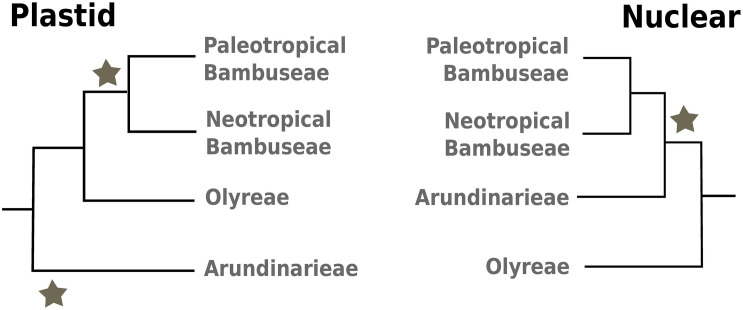
Phylogenetic relationship of the bamboo clades. The figure summarizes established phylogenetic relationships between the herbaceous Olyreae and the three woody clades: Arundinarieae, Paleotropical, and Neotropical Bambuseae. The two panels represent the tree topologies based on plastome **(left)** or nuclear **(right)** analyses. The gray stars indicate the woody lineages. They show that nuclear analyses support the monophyly of the woody bamboos (versus their paraphyly in plastome analyses).

The DNA data from individual plastid markers or plastid genomes (plastomes) unambiguously support Bambuseae as sister to the Olyreae, with Arundinarieae sister to that clade, rendering the woody bamboos paraphyletic and demonstrating that the Bambuseae and Olyreae share a plastome (Figure 1, left; Sungkaew et al., 2009; Kelchner and Bamboo Phylogeny Group, 2013; Wysocki et al., 2015; Zhang et al., 2016). Within the primarily Asiatic Arundinarieae, plastome sequence data resolve 12 numbered lineages or clades (I-XII), classified into five subtribes (Triplett and Clark, 2010; Zhang et al., 2012, 2016, 2020; Yang et al., 2013; Attigala et al., 2016). All Arundinarieae for which counts are available share the same chromosome number (2n = 48) and the tribe is considered to be tetraploid (Soderstrom, 1981; Hilu, 2004). Within Bambuseae, plastome sequence data support two major sister clades, the Paleotropical woody bamboos (PWB; 554 species in eight subtribes) and the Neotropical woody bamboos (NWB; 446 species in three subtribes), although support for the monophyly of the NWB is less robust than for the PWB (Kelchner and Bamboo Phylogeny Group, 2013; Wysocki et al., 2015; Zhang et al., 2016). The Bambuseae show a range of chromosome counts, but tetraploidy is inferred for the NWB clade and hexaploidy for the PWB clade (Soderstrom, 1981; Li et al., 2001; Hilu, 2004). Within Olyreae, the monotypic New Guinean endemic *Buergersiochloa* is strongly supported as sister to the wholly Neotropical subtribes Parianinae and Olyrinae (with the exception of one weedy species that also occurs in Africa) (Kelchner and Bamboo Phylogeny Group, 2013; Oliveira et al., 2014; Clark et al., 2015; Wysocki et al., 2015; Zhang et al., 2016). The Olyreae are fundamentally diploid, and instances of polyploidy appear to be of relatively recent origin (Judziewicz et al., 1999; Triplett et al., 2014).

In contrast, although based on relatively sparse sampling or data, the nuclear signal across the Bambusoideae supports monophyly of the woody bamboos (Arundinarieae + Bambuseae) with Olyreae sister to the woody clade (Figure 1, right; Triplett et al., 2014; Wysocki et al., 2016; Guo et al., 2019). Triplett et al. (2014) analyzed sequences for three low-copy nuclear markers (*gpa1*, *pabp1*, *pvcel1*) and provided evidence for two and possibly three ancient hybridization events leading to allopolyploidy in the Arundinarieae and the Bambuseae. Separate A and B genomes represented the putative diploid ancestors of the Arundinarieae that led to an allotetraploid progenitor, which underwent subsequent diversification. Triplett et al. (2014) also hypothesized that genomes C and D were united through another ancient hybridization event leading to allotetraploidy in a tropical woody bamboo ancestor (or the NWB clade). In their model, genome E then hybridized with the tetraploid tropical woody bamboo ancestor (or a member of the NWB clade), leading to the hexaploid PWB. Wysocki et al. (2016) generated floral transcriptomes for four bamboo species representing the three tribes, and also recovered a woody bamboo clade. They hypothesized that whether woody bamboos are monophyletic or not, a minimum of one ancient hybridization event (between a progenitor of Olyreae and a progenitor of Bambuseae) is needed to explain the plastid signal shared between these two lineages.

Guo et al. (2019) analyzed the nuclear genomes of four bamboo species representing the Arundinarieae, Bambuseae (one each of the PWB and NWB clades) and Olyreae. Based on 61 ‘perfect-copy’ syntenic genes common to woody bamboos, they estimated a divergence time between the progenitor of Olyreae and that of the woody bamboos of ∼42 mya (million years ago), and a divergence time between the progenitors of the Bambuseae and Arundinarieae of ∼33 mya. They inferred four extinct diploid ancestors of the woody bamboos (genomes A, B, C, and D) and three ancient allopolyploid events leading to the Arundinarieae (CD), Neotropical Bambuseae (BC) and Paleotropical Bambuseae (ABC), respectively. Notably, they proposed that all extant woody bamboos share the C genome. The WGDs in these three lineages were estimated at 19-20 mya, somewhat earlier than the 7–12 mya estimate for Arundinarieae of Peng et al. (2013). Except for the fifth genome/diploid ancestor postulated by Triplett et al. (2014), the scenarios of Triplett et al. (2014) and Guo et al. (2019) are essentially the same, and both fail to explain the plastome shared by Bambuseae and Olyreae.

All flowering plant (angiosperm) genomes are descended from lineages that underwent WGDs, often several times, over the last 300 million years (Paterson et al., 2004; Cui et al., 2006; Soltis et al., 2009; Jiao et al., 2011; Escudero and Wendel, 2020). Following polyploidization, genomes tend to reach a more stable diploid state through a process called diploidization. Diploidization is routinely associated with chromosomal rearrangements that are expected to impair homolog pairing and the reduction of gene content through an accumulation of often tiny deletions that remove some gene copies resulting from the whole genome duplication (Ilic et al., 2003; Conant et al., 2014). Often, but not always (VanBuren et al., 2020) this gene loss, called fractionation, is biased toward gene removal from a particular parental genome (Thomas et al., 2006; Bird et al., 2018; Wendel et al., 2018). While it is not clear that biased fractionation during diploidization is a general rule, more and more cases support the phenomenon, including genomes of maize, wheat, cotton, Brassica and Arabidopsis (Schnable et al., 2011; Woodhouse et al., 2014; Renny-Byfield et al., 2015; Pont and Salse, 2017). Epigenetic gene silencing and transposable element (TE) (mobilome) proliferation/silencing are believed to be major players during the genome dominance and biased fractionation process (Wendel et al., 2018).

A powerful tool for understanding the evolution of genomes is comparison across a well-characterized phylogenetic tree. The strategy referred to as “sample sequencing analysis” (SSA) can provide the raw data both for nuclear genome repeat analysis and for plastid-based phylogenetic characterization (Brenner et al., 1993; Devos et al., 2005; Estep et al., 2012, 2013). All plant genomes contain abundant repeats, primarily TEs (Bennetzen, 2000; Devos, 2010; Bennetzen and Wang, 2014), with exceptional abundance of long terminal repeat- (LTR-) retrotransposons particularly common. Genome sample sequencing at low depths can describe in depth any repeat that is present at > 50X in the data set, for instance as with 0.1 X genome coverage of a repeat that has a copy number > 500X (Park et al., 2021). Because plastid genomes are present in thousands of copies in any leaf cell, for instance, a plastid genome assembly is also routinely feasible from such SSA data.

The well supported but conflicting plastid and nuclear signals in the bamboos provided an opportunity to test the utility of SSA data in illuminating the evidently complex evolutionary history of this major lineage of grasses. We generated SSA data for 51 bamboo species representing all three recognized tribes and estimated their genome sizes using flow cytometry. We assembled plastomes for the 51 species and analyzed repeat (mobilome) diversity in their nuclear genomes. Leveraging the plastid and nuclear genomes simultaneously from the same set of samples allowed us to formulate a synthetic hypothesis of bamboo evolution that incorporates processes associated with WGD events and explains the apparent plastid vs. nuclear conflict. We confirm the roles of ancient hybridization and allopolyploidy in the evolutionary history of bamboos, but also propose that bamboos represent yet another example of biased fractionation and that some lineages within the woody bamboos have undergone diploidization. We conclude with suggestions for strategies to test this hypothesis.

## Materials and Methods

### Construction of the Bamboo Data Sets

Sampling was designed to represent all three bamboo tribes, including as many of the numbered lineages as possible from the Arundinarieae, and as many subtribes as possible from the Bambuseae and Olyreae, based on plant material available in the United States. Five living collections were visited to obtain the samples: Bamboo Garden Nursery (North Plains, Oregon); Pohl Conservatory, Iowa State University (Ames, Iowa); Tradewinds Bamboo Nursery (Gold Beach, Oregon); Tropical Bamboo Nursery and Gardens (Loxahatchee, Florida); and the USDA-ARS Fruit and Nut Tree Station (Byron, Georgia). Multiple accessions of a few taxa (e.g., *Neohouzeaua mekongensis*, *Ampelocalamus scandens*, *Chusquea* spp.) were targeted to test identifications and the resolving power of the plastome data. Of the 51 bambusoid taxa (i.e., excluding the two non-bambusoid outgroups sampled for this study), Arundinarieae is represented by 17 species from six of the 12 numbered lineages representing three of five subtribes, Bambuseae is represented by 19 Paleotropical species representing four of eight subtribes and by nine Neotropical species representing all three subtribes, while Olyreae is represented by six species representing two of the three subtribes (Table 1). Because it was not possible to voucher all of the samples due to time limitations, we conducted a comparative plastome analysis to confirm identifications to the extent possible. For the comparative 115 Bambusoideae plastome analysis, an additional 64 plastomes (33 Arundinarieae, 21 Bambuseae, and 10 Olyreae) were downloaded from GenBank (Supplementary Table 1) and were analyzed with the 51 plastomes generated for this study, for a total of 99 different ingroup species (non-redundant species). For both the 51 and 115 taxon data sets, *Zizania aquatica* (Oryzoideae) and *Lolium multiflorum* (Pooideae) were used as the outgroup taxa.

**TABLE 1:** Taxa sampled for the 51 plastome and repeat analyses.

Taxon	Abbreviation	Voucher or living collection and plot number
**Olyreae (herbaceous bamboos)**		
*Diandrolyra* sp.	Dspe	Clark 1301 (ISC)
*Eremitis* sp.	Espe	Clark and Zhang 1343 (ISC)
*Lithachne pauciflora*	Lpau	Clark 1297 (ISC)
*Pariana radiciflora*	Prad	Pohl s.n. (ISC 412133)
*Raddia brasiliensis*	Rbra	Clark and Attigala 713 (ISC)
*Raddia distichophylla*	Rdis	Clark and Zhang 1306 (ISC)
**Arundinarieae (temperate woody bamboos)**		
*Ampelocalamus scandens* IA	AscI	Clark 1291 (ISC)
*Ampelocalamus scandens* FL	AscF	TBNG 2
*Arundinaria gigantea* ‘Macon’	Agig	BGN 1
*Bergbambos tessellata*	Btes	BGN
*Chimonocalamus pallens*	Cpal	BGN
*Drepanostachyum khasianum*	Dkha	TBNG 151
*Fargesia denudata*	Fden	BGN 19
*Fargesia murielae*	Fmur	BGN 23
*Fargesia robusta* ‘Campbell’	Frob	BGN 25
*Oldeania alpina*	Oalp	TBN/Attigala and Clark 170 (ISC)
*Phyllostachys bambusoides*	Pbam	BGN 49
*Phyllostachys edulis* ‘Jaquith’	PedJ	BGN
*Phyllostachys heteroclada*	Phet	BGN 61
*Pleioblastus simonii*	Psim	BGN
*Pseudosasa cantorii* Plot B-7	PcaB	Soderstrom 2501 (US); Clark and Hotchkiss 1339 (ISC)
*Sasa veitchii*	Svei	BGN 95
*Shibataea kumasaca*	Skum	BGN 101
**Bambuseae (tropical woody bamboos)**		
*Bambusa atra*	Batr	TBNG 4
*Bambusa bambos*	Bbam	TBNG 6
*Bambusa chungii*	Bchu	TBNG 12
*Bambusa multiplex* ‘Alphonse Karr’	Bmuk	TBNG 41
*Bambusa oldhamii*	Bold	TBNG 53
*Bambusa variostriata*	Bvar	TBNG 91
*Cephalostachyum pergracile*	Cper	TBNG 99
*Cephalostachyum scandens*	Csca	TBNG 101
*Chloothamnus (Nastus) elatus*	Cela	TBNG 191/Clark and Ruiz 1706
*Chusquea circinata*	Ccir	TBN
*Chusquea circinata* ‘Chiapas’	CciC	TBN
*Chusquea culeou* ‘Hillier’	Ccuh	BGN
*Chusquea culeou* Aisen II	CcuA	BGN
*Chusquea gigantea*	Cgig	TBN
*Dendrocalamus giganteus*	Dgig	TBNG 121
*Dendrocalamus strictus*	Dstr	TBNG 146
*Dinochloa malayana*	Dmal	TBNG 150 (sight verified by LGC)
*Gigantochloa atter*	Gatt	TBNG 156
*Gigantochloa hasskarliana*	Ghas	TBNG 159
*Guadua angustifolia*	Gang	TBNG 181
*Guadua paniculata*	Gpan	TBN
*Neohouzeaua mekongensis* FL	NmeF	Fairchild Plot 159
*Neohouzeaua mekongensis* IA	NmeI	Clark and Attigala 1712
*Neololeba atra*	Natr	TBNG 190
*Otatea acuminata aztecorum*	Oazt	Clark and Zhang 1348 (ISC)
*Oxytenanthera abyssinica*	Oaby	Clark and Triplett 1664 (ISC)
*Rhipidocladum pittieri*	Rpit	Clark and Zhang 1349 (ISC)
*Schizostachyum brachycladum*	Sbra	TBNG 199

*BGN, Bamboo Garden Nursery; TBGN, Tropical Bamboo Garden and Nursery; TBN, Tradewinds Bamboo Nursery; ISC, Ada Hayden Herbarium at Iowa State University.*

### DNA Extraction and Sequencing

Collected samples were maintained in cold conditions at –80°C. For DNA extraction, 0.05–0.08 g of fresh leaves (weighed before freezing) were used. Disruption of tissues was done using a TissueLyser II. Lysis steps of DNA extraction were performed using Plant DNAzol-ES reagents (MRC catalog #DN128) and finally Qiagen DNeasy Plant Mini Kits (Qiagen catalog no. 69104). Quantity and quality were checked by Nanodrop and 1% agarose gel migration, respectively. Illumina technology, NextSeq (300 cycles) paired-end 150 bp High Output Flow Cell (FastQ files, project accession PRJEB43575), at the Georgia Genomics Facility (GGF^1^) was used for sequencing. All samples were sequenced on a single lane to obtain low-depth sequence data. Coverages were calculated as follows: C = LN/G, where L corresponds to the length of the reads, N is the number of reads and G is the haploid genome size. Coverages ranging from 0.5X (*Phyllostachys heteroclada*) to 5.1X (*Raddia distichophylla*) with a median value of 1.26 were obtained.

### Phylogenetic Estimation Based on Plastomes

Plastome reconstructions were performed as described in Wysocki et al. (2014). Fastq files were trimmed to remove short reads (<25 bp in length). SPAdes 3.5.0 (Bankevich et al., 2012) was used with k-mer lengths of 19–85 increasing in steps of six, to perform *de novo* assemblies of each set of reads into contigs. Anchored conserved region extension was used to locate the largest plastome contigs and move them to the correct place in the genome. This method uses regions that are ∼100% conserved among grasses and information on their locations in the plastome. Next, contigs were extended using either genome walking or reference mapping until the adjacent contig was reached (with > 20 bp overlap). The boundaries of the major inverted repeat regions were located following the method of Burke et al. (2012). Annotations were then transferred from a closely related and previously published plastome based on sequence similarity using Geneious Pro (Biomatters Ltd., Auckland, New Zealand). All protein coding sequences were inspected for in frame start and stop codons. Multiple plastome sequence alignment was realized using MAFFT 7.187 (Katoh et al., 2002) after which all positions with gaps introduced by the alignment were removed.

Phylogenetic reconstructions were performed through two different methods, both giving similar results. MrBayes^2^ was used for Bayesian inference (BI) reconstructions with 1,000,000 generations and sampled every 100 generations. Maximum likelihood (ML) reconstructions -shown in the result section- were done using RaxML-NG using –all –model GTR + G options (Kozlov et al., 2019). Models of character evolution were compared (jModelTest v2-1.10; Darriba et al., 2012) under the Akaike information criterion (Akaike, 1974). The GTR + G model was among the best-fit models and was specified for the plastome phylogenomic analysis. The –all is an “all-in-one” analysis that will automatically search for both the best ML tree and bootstrap replicates for this tree (20 ML trees and 100 bootstraps) (Kozlov and Stamatakis, 2020). Phylogenetic estimations were performed on both the 51 and 115 taxon data sets. Posterior probability (PP) and Maximum Likelihood (ML) bootstrap (MLBS) support values were calculated for the Bayesian Inference (BI) and ML analyses, respectively. *Zizania aquatica* (Oryzoideae) and *Lolium multiflorum* (Pooideae) were used as the outgroup taxa for all phylogenetic reconstructions.

### Repeat Analyses

Repeat diversity, including interspersed elements and simple repeats, was investigated in the newly sequenced species. Adapters and quality trimming were performed on fastq files using Trim Galore! version 0.4.0^3^ using parameters –length 45 –paired –trim 1 and default quality 20. RepeatExplorer (Novák et al., 2013) was used to investigate repeat diversity with the implemented default universal RepeatMasker library 4.0.6 and option -f (clustering with comparative analysis based on species prefix). RepeatExplorer recognizes reads belonging to a family to make a cluster. Per definition, a cluster is a repeat family with shared > 80% sequence identity and different repeat families are clusters that share less than 80% identity (Wicker et al., 2007). The Venn diagram and correlation matrix were realized in the R environment using, respectively, “VennDiagram” and “pheatmap” packages (R Core Team, 2019).

### Genome Size Estimation

Genome sizes were estimated using flow cytometry at the Benaroya Research Institute (Seattle, WA, United States) from fresh young leaves following the protocol of Arumuganathan and Earle (1991). For each species, the experiment was conducted with four replicates. *Oryza sativa* (rice, Oryzoideae) was used as the genome size reference. Genome size data are summarized in Supplementary Table 2.

## Results

### Plastome Analyses

Assemblies were performed for 51 newly generated plastomes for this study. After saving only one copy of the large inverted repeat region, the full plastome alignment was 140,571. In comparison across all 51 plastomes, this alignment was reduced to 95,761 bp after removal of gapped positions.

**FIGURE 2:**
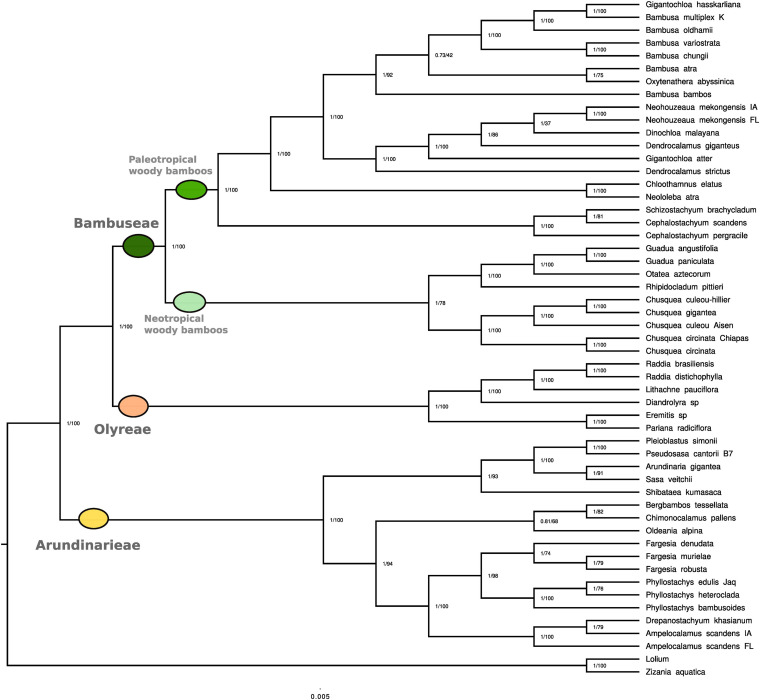
Phylogenetic reconstruction based on full plastome sequences. Phylogenetic reconstruction using whole plastid genomes representing 51 bamboo species. Two non-bamboo plastomes were added as outgroups (*Lolium multiflorum* and *Zizania aquatica*). Among the 51 collected species, plastome data were already available for 17 of them. Values represent Bayesian posterior probability and maximum likelihood bootstrap scores, respectively.

In both BI and ML analyses, Bambusoideae and each of the three tribes were fully supported as monophyletic (PP 1.00, MLBS 100, hereafter in this order) (Figure 2). The sister relationship of Arundinarieae to the Bambuseae + Olyreae clade also received support values of PP 1.00 and MLBS 100.

The taxa representing six numbered lineages of Arundinarieae were recovered in two major clades. One of these (1.00, 93) comprised the representatives of lineages IV (*Shibataea kumasaca*) and VI [(*Sasa veitchii* + *Arundinaria gigantea*) + (*Pseudosasa cantorii* + *Pleioblastus simonii*)] with IV sister to VI. The other (1.00, 94) comprised the representatives of lineage V (*Ampelocalamus* through *Fargesia* in Figure 2) forming a clade sister to a clade formed by lineage II (*Oldeania alpina*) + [lineages I (*Bergbambos tessellata*) + III (*Chimonocalamus pallens*)]. Support values for internal relationships within lineage VI were generally high whereas those within lineage V were more variable (Figure 2). Both *Fargesia* and *Phyllostachys* were recovered as monophyletic, with PP 1.00 and MLBS 74 and 100, respectively. The two accessions of *Ampelocalamus scandens* (from Florida and Iowa) were supported in the same clade as *Drepanostachyum khasianum* (1.00, 100), but the Iowa accession was recovered as sister to *D*. *khasianum* (1.00, 79).

Within the Olyreae, the two sampled subtribes, Parianinae and Olyrinae, were each supported as monophyletic (1.00, 100). Within the Olyrinae, the two species of *Raddia* were sister (1.00, 100) with *Lithachne pauciflora* sister to *Raddia* (1.00, 100) and *Diandrolyra* sp. sister to the *Raddia* + *Lithachne* clade.

The sampled taxa of Bambuseae were recovered in two major clades, the PWB (1.00, 100) sister to the NWB (1.00, 78). Within the NWB, the Chusqueinae were supported as monophyletic (1.00, 100) and sister to the Arthrostylidiinae + Guaduinae clade (1.00, 100). Within Chusqueinae, the two accessions of *Chusquea circinata* were sister (1.00, 100) and the three Chilean accessions formed a clade (1.00, 100). However, the *C. culeou* Hillier accession was strongly supported (1.00, 100) as sister to *C. gigantea*, with *C. culeou* Aisen sister to that clade. The single accession of Arthrostylidiinae (*Rhipidocladum pittieri*) was sister to a monophyletic Guaduinae (1.00, 100), and *Guadua* was supported as monophyletic (1.00, 100).

Three representatives of subtribe Melocanninae formed a strongly supported clade (1.00, 100) sister to the remaining PWB. *Neololeba atra* (Dinochloinae) and *Chloothamnus elatus* (Racemobambosinae) were strongly supported as sister (1.00, 100) and this clade was strongly supported as sister (1.00, 100) to the representatives of Bambusinae plus *Dinochloa malayana* (Dinochloinae) and the two accessions identified as *Neohouzeaua mekongensis* (Melocanninae). All species of *Bambusa* formed a clade with *Oxytenanthera abyssinica* and *Gigantochloa hasskarliana* (1.00, 92), which was sister to the clade (1.00, 100) formed by the two species of *Dendrocalamus*, *G. atter*, *D. malayana* and the two *Neohouzeaua* accessions. Internal relationships within the two Bambusinae clades were mostly moderately to strongly supported, although two nodes received < MLBS 50 and one of those had a PP of 0.73.

Initial alignment of the 115 plastome sequences was 166,221 bp in length and reduced to 95,763 bp. The 115-plastome analysis also fully supported the monophyly of the Bambusoideae and each of the three tribes, as well as the Arundinarieae + (Bambuseae + Olyreae) topology (all with MLBS 100) (Supplementary Figure 1). Eighteen of the newly generated plastomes were for species with existing plastomes; seven were from the same voucher and 11 were from different vouchers. With two exceptions (*Pariana radiciflora* and *Guadua angustifolia*), the existing and new plastomes for any given species were resolved as sister to each other, 15 of the pairs with MLBS 100 and one (*Bambusa oldhamii*) with MLBS 96. In the case of *P. radiciflora*, the new plastome was resolved as sister to the clade [*Pariana* sp. + (*P. campestris* + *P. radiciflora*)], all nodes with MLBS 100. The new plastome of *G. angustifolia* was resolved as sister to *G. weberbaueri* (MLBS 70) with the existing plastome of *G. angustifolia* sister to that clade (MLBS 66). All lineages of Arundinarieae for which more than one taxon was sampled were resolved as monophyletic; lineages I, II, VII, IX, and XI were each represented by a single species even if two accessions were analyzed (as for I and II). Lineage XI (*Hsuehochloa calcarea*) was strongly supported as sister to the remaining Arundinarieae (MLBS 100).

Within the Olyreae, the Olyrinae and Parianinae were resolved as sister (MLBS 100) with the monotypic Buergersiochloinae sister to that clade (MLBS 100). All other internal relationships within the Olyreae received support values of MLBS 100.

Within the Bambuseae, the NWB clade received a support value of MLBS 47, with the Chusqueinae sister to Arthrostylidiinae + Guaduinae, and the PWB clade was strongly supported (MLBS 100). Four taxa representing the Melocanninae formed a well-supported clade (MLBS 100) sister to the remaining PWB. A strongly supported clade (MLBS 100) consisting of all Bambusinae representatives plus *Dinochloa malayana* (Dinochloinae) and the two accessions identified as *Neohouzeaua* were resolved as sister to a clade (MLBS 92) consisting of *Neololeba* (Dinochloinae) + *Greslania* sp. (Greslaniinae) sister to *Chloothamnus* (Racemobambosinae) + *Hickelia* (Hickeliinae). Within the Bambusinae clade, all representatives of *Bambusa* plus *Oxytenanthera* and *Gigantochloa hasskarliana* formed a clade (MLBS 96) sister to a strongly supported clade (MLBS 100) consisting of two species of *Dendrocalamus*, *G. atter*, *D. malayana* and the *Neohouzeaua* accessions.

### Repeat Analyses Using SSA Data

Repeat diversity was investigated as representative of the nuclear genome. Repeats contain both interspersed elements, including TEs (mostly LTR-retrotransposons in angiosperms), and simple repeats such as satellite sequences. To better understand the relationships among Olyreae, Arundinarieae and the two Bambuseae lineages, the Paleotropical and Neotropical Bambuseae were treated as two different clades in these analyses.

A total of 655,417 clusters was detected in bamboo sequences, with 365 clusters making up to 50% of the total number of clustered reads and 23 clusters making up to 10% of the total number of clustered reads. Of these 365 clusters, 134 belong to Gypsy retrotransposons, 94 belong to Copia retrotransposons, 65 to DNA transposons, 64 to simple repeats and 8 to other types of repeats. All top 10% most abundant repeats belong either to Gypsy or Copia retrotransposons.

**FIGURE 3:**
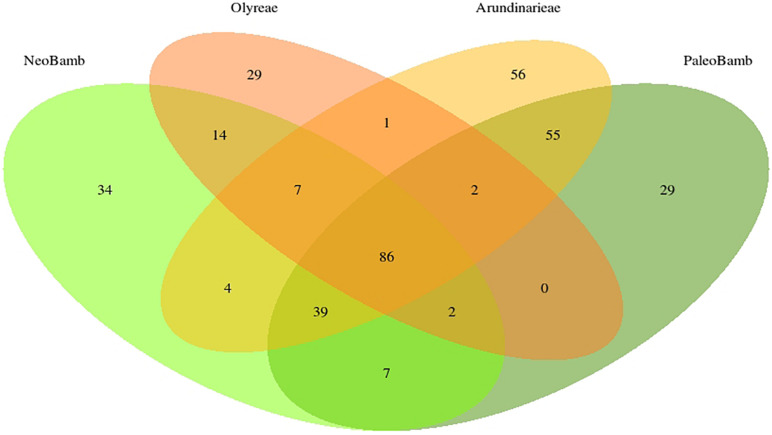
Number of species-specific and shared repeat TE clusters in the bamboo clades. Only the 365 most abundant clusters were used to draw the Venn diagram. Out of these 365 clusters, 86 are found in all four of the studied clades.

Of these 365 clusters, 86 are common to the four clades; 141 clusters are present in the Olyreae, 250 in the Arundinarieae, 193 in the Neotropical Bambuseae and 220 in the Paleotropical Bambuseae (Figure 3). The Olyreae shares 1 cluster with Arundinarieae, 0 with the Paleotropical and 14 with the Neotropical Bambuseae (clusters shared by only these two clades). Similarly, the Arundinarieae shares 4 clusters with the Neotropical Bambuseae and 55 with the Paleotropical Bambuseae. And, finally, the two Bambuseae clades share 7 clusters. Arundinarieae and the two Bambuseae clades (the woody bamboos collectively) have 39 common clusters.

**FIGURE 4:**
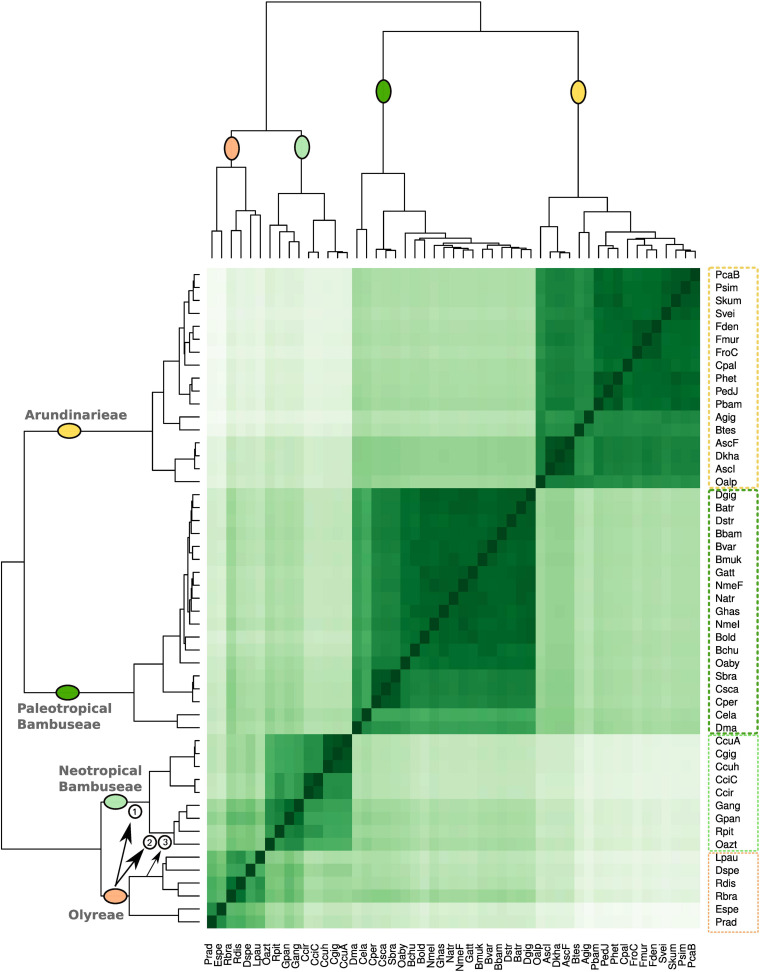
Bamboo species similarity based on repeat diversity. The correlation matrix was built using the 365 most abundant repeat clusters using pheatmap R function. Upper and left sides represent the same phylogenetic relationship. Three proposed hybridization events between Olyreae and Neotropical woody bamboos are highlighted with the arrows and numbers. These events are then depicted in Figures 6A,B.

To investigate the relationships among the bamboo clades based on repeat cluster diversity, a correlation matrix was built using the 365 most abundant repeat clusters of the 51 studied species (Figure 4). The 51 species are distributed among the four distinct clades: Olyreae, Arundinarieae, and the Neotropical and Paleotropical Bambuseae. The Olyreae share a common branch with the Neotropical Bambuseae, whereas Arundinarieae group with the Paleotropical Bambuseae.

Although the four clades were recovered as four groups, clustering of taxa within each group was not necessarily consistent with the plastome signal. Within Olyreae, the two deepest branches correspond to the Parianinae and Olyrinae, as seen in the plastome topology; the only difference is that *Diandrolyra* and *Lithachne* form a cluster instead of forming serial clusters to the *Raddia* cluster. Within the Neotropical Bambuseae cluster, the two deepest branches correspond to the Chusqueinae and the Arthrostylidiinae + Guaduinae clade, and the main difference is that the positions of *Rhipidocladum* and *Otatea* are transposed relative to the plastome topology, so that the Guaduinae do not form their own cluster. Within the Arundinarieae and the Paleotropical woody bamboo groups, however, the matrix resolution largely does not reflect the plastome topology. *Nastus elatus* and *Dinochloa malayana* form one of the two deepest branches of the Paleotropical Bambuseae, with all remaining species in the other branch. The three species of the subtribe Melocanninae (*C. pergracile*, *C. scandens*, and *S. brachycladum*) cluster together, but the remainder of the taxa are intermixed. Within the Arundinarieae, congeneric species (i.e., from *Phyllostachys* or *Fargesia*) group together, as do *Ampelocalamus* and *Drepanostachyum* in one cluster and *Pleioblastus*, *Pseudosasa*, *Sasa*, and *Shibataea* in another. Otherwise the taxon clusters do not match any pattern predicted by the plastome topology.

While the repeat clustering unambiguously distributed species among four distinct taxon clusters corresponding to the four clades, smaller repeat clusters can be distinguished. Within the Neotropical Bambuseae, one cluster shows that repeats are shared between Olyreae and the Arthrostylidiinae + Guaduinae clade (Figures 2, 4) and a second smaller cluster shows that repeats are shared between Olyrinae specifically (Lpau, Dspe, Rdis, and Rbra) and the Arthrostylidiinae + Guaduinae clade (Figures 3, 4).

### Genome Size Estimations

**FIGURE 5:**
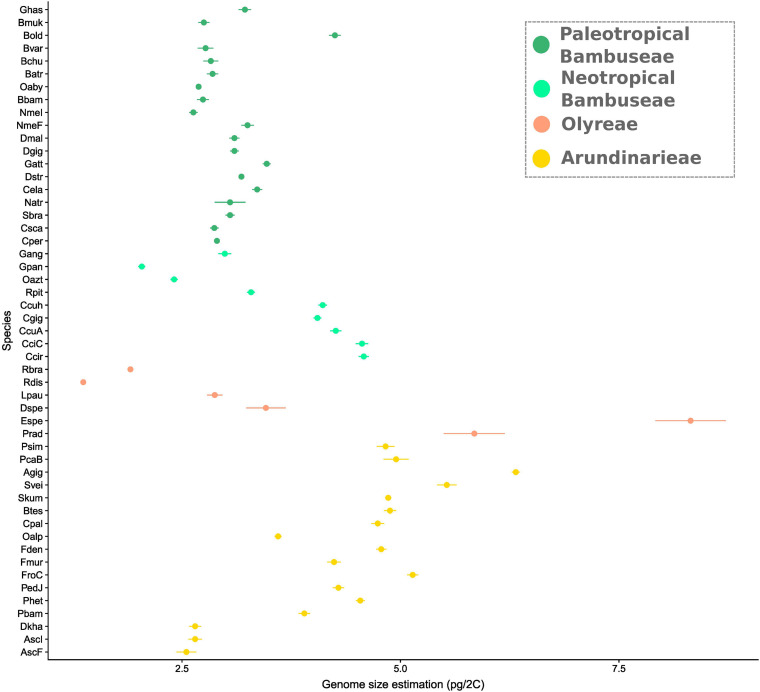
Genome size variation in bamboo. Estimations were done using flow cytometry. Error bars represent standard deviations. Dots are clade-colored with yellow showing temperate bamboos, orange dots are herbaceous, light green is Neotropical woody and dark green is Paleotropical woody. Taxa are ordered along the *Y* axis in the phylogenetic relatedness order depicted in Figure 2.

Genome sizes varied from 1.37 to 8.32 pg/2C (*R. distichophylla* and *Eremitis* sp. respectively, both herbaceous species) (Supplementary Table 2), with a mean size of 3.69 pg/2C (Figure 5). Within the Arundinarieae, genome size varied from 2.55 (*Am. scandens*) to 6.32 (*A. gigantea*) pg/2C with a mean and a median size of 4.38 and 4.74, respectively. The Olyreae displayed the most heterogeneous values, with the minimum and maximum of all species, as mentioned. PWB displayed relatively homogeneous genome sizes from 2.63 (*N. mekongensis* from the Iowa State collection) to 3.47 (*G. atter*), except the outlier *B. oldhamii* at 4.25 pg/2C. Last, the smallest genome size within the NWB was 2.04 pg/2C (*G. angustifolia*) and the largest was 4.58 (*C. circinata*). More specifically, the Chusqueinae harbored a higher range from 4.05 to 4.58 pg/2C (mean size: 4.31 pg/2C) than the Arthrostylidiinae + Guaduinae (from 2.04 to 3.29 pg/2C, mean size: 2.68 pg/2C).

## Discussion

Polyploidy is very frequent in plant genomes, and the grass family (Poaceae) exemplifies this trend (Kellogg, 2015). The Poaceae underwent a whole genome duplication (WGD) event early in its evolution and multiple other WGDs occurred during its diversification, including in the bamboos (Bambusoideae) (Paterson et al., 2004; Yu et al., 2005; Wang et al., 2015; Escudero and Wendel, 2020). Ancient hybridization and polyploidy presumably played a major role in the evolution of the woody bamboos, and it is also reasonable to assume that genome dominance (biased fractionation) and diploidization have occurred (Escudero and Wendel, 2020), but bamboo evolution has not been comprehensively interpreted in light of these processes. We here synthesize evidence from plastome phylogenetics, repeat diversity and genome size data, and propose an evolutionary scenario for the bamboos that incorporates biased fractionation and diploidization in addition to ancient hybridization and polyploidy, thus accounting for the apparently contradictory results of prior studies. For the sake of clarity and simplicity, we first discuss our results and then we interpret the evolutionary history of the bamboos based on our results.

### Plastome Signal Is Consistent With Prior Analyses

The overall topology generated in the 51-plastome analysis (Figure 2) is completely consistent with prior plastid-based studies (Sungkaew et al., 2009; Kelchner and Bamboo Phylogeny Group, 2013; Wysocki et al., 2015; Zhang et al., 2016) in resolving a strongly supported sister relationship between Bambuseae and Olyreae, with Arundinarieae sister to that clade. Within Bambuseae, a PWB clade and an NWB clade were resolved, with strong support and less robust support, respectively, also as seen in the studies cited above. Except for the placements of *Neohouzeaua mekongensis* and *Dinochloa malayana* (PWB), relationships reflect those recovered in other studies focused on either the PWB or the NWB (e.g., Sungkaew et al., 2009; Kelchner and Bamboo Phylogeny Group, 2013; Zhou et al., 2017b). Likewise, internal relationships within Olyreae and Arundinarieae are consistent with prior studies (e.g., Zhang et al., 2016; Ferreira et al., 2019).

The 115-plastome analysis also fully supported the monophyly of the Bambusoideae and each of the three tribes, as well as the Arundinarieae + (Bambuseae + Olyreae) topology (Supplementary Figure 1). With a few exceptions, the existing and new plastomes for any given species were resolved as sister to each other, as expected, and even the exceptions were placed within the same clade as the plastome of the corresponding species. All other newly generated plastomes, except those of *N*. *mekongensis* and *D*. *malayana*, fell within the clades as predicted based on prior analyses (Sungkaew et al., 2009; Kelchner and Bamboo Phylogeny Group, 2013; Wysocki et al., 2015; Zhang et al., 2016; Zhou et al., 2017b; Ferreira et al., 2019), confirming the identifications of the samples used in the present study.

The two samples identified as *Neohouzeaua mekongensis*, one from the Iowa State University collection and one from the Fairchild Tropical Garden, represent the same introduced taxon, and possibly the same introduction. However, the anomalous placement of the two samples indicated that this material is misidentified, as *Neohouzeaua* is classified within the PWB subtribe Melocanninae, and these samples nested within the Bambusinae. Although no definitive identification has been made, the material likely represents a species of *Gigantochloa* (S. Dransfield, personal communication) and thus the corresponding voucher identifications will be changed. *Dinochloa*, on the other hand, is expected to group with *Neololeba atra* of the PWB, but the sample of *D*. *malayana* was resolved as sister to the *Neohouzeaua* samples (51-plastome topology) or sister to the newly generated plastome of *Dendrocalamus giganteus* in a clade sister to the *Neohouzeaua* samples (115-plastome topology). In both instances, ML bootstrap support values were extremely low, although the posterior probability in the 51-plastome topology was 1.00. The plant growing at the Tropical Bamboo Nursery and Gardens was confirmed as *D*. *malayana* by L. Clark, so potential explanations include possible contamination of DNA samples or chimeric plastomes.

### Repartition Analysis From Sample Sequence Data in the Bamboos

Sample sequence analysis (SSA) uncovers the major genome repeat content in a genome in a very short time, at a very low cost and with very robust analytical power (Estep et al., 2012; Park et al., 2021). Larger genome sizes actually facilitate this analysis, because the higher copy number repeats in genomes are often more easily identified with lower genome sequence redundancy. Ploidy level does not affect data generation or analysis in any way vis-à-vis describing the repeat content. The only limitation to the SSA technique is the inability to describe low copy number sequences in any comprehensive manner, but our goal was purely description of the most abundant repeats (and thus those contributing most to genome content/size in all known grasses, including the bamboos).

Repartition analysis, in contrast, is not routinely applied to analysis of genomic repeats. This powerful technology does not ascribe any particular pre-conceived value to any repeat family or families. The total major repeat content in each genome is analyzed, and any signals are mined from the observed comparative results. In our study, the repartition analysis confirmed our results for terminal branches with plastid markers, thereby demonstrating its phylogenetic power. However, most important (as described below), the partition analysis suggested a history of wide interactions (e.g., ancient crosses) to create transient polyploids from distantly related parents that, thereby, provides insights into the basal origin of woody, tropical and temperate bamboos. This is a novel use of repeats, of SSA, and of partition analysis.

### Repetitive DNA Signal Differs From the Plastome Signal

The study of repeats (TEs and other interspersed repeats) has provided fundamental answers regarding genome evolution and history in many published studies (reviewed in Bennetzen and Wang, 2014). However, like genome size, TE and other repeat content can more than double in just a million years or two even without polyploidy (Estep et al., 2013), and many of these changes could be horizontal DNA transfers (Park et al., 2021), so neither genome size nor TE content has proven consistently useful as a phylogenetic signal (Kellogg and Bennetzen, 2004). However, rather than following individual TE families, superfamilies or overall genome size, it is possible that the overall TE population characteristics in a genome might indicate properties of nuclear genome history. In this regard, our TE data (but not genome sizes) for each genome were fully compatible with the nuclear phylogenetic history derived by nuclear gene analyses in the bamboos (Triplett et al., 2014; Wysocki et al., 2016; Guo et al., 2019), thus confirming that repeats are a useful confirmatory phylogenetic indicator in this particular set of genomes.

In our repeat analyses, homology studies indicated that the great majority of the most abundant repeat clusters in the bamboos are TEs, so we carefully inspected the TE data. Making up more than half of most plant genomes (e.g., in grasses, >80% of the maize genome, Schnable et al., 2009; almost 60% of the moso bamboo genome, Peng et al., 2013) and with families often found in high copy numbers, TEs are easily detectable in low coverage sequencing data (Park et al., 2021). They are considered to be major drivers of genome rearrangement, including the evolution of novel gene regulation, especially epigenetic gene regulation (Lisch and Bennetzen, 2011). TE-associated changes in gene regulation are observed during the genome fractionation and diploidization processes (Freeling et al., 2012; Renny-Byfield et al., 2015; Vicient and Casacuberta, 2017; Bird et al., 2018; Wendel et al., 2018). On some occasions, TEs have been observed to be horizontally transmitted (El Baidouri et al., 2014; Park et al., 2021). However, they are usually vertically transmitted, making them potentially useful phylogenetic markers (Deragon and Zhang, 2006). While some studies use repeat abundance (Dodsworth et al., 2015) or repeat family identities (Vitales et al., 2020), we investigated bamboo lineage relatedness based on the repartition (potential to share clusters between species and lineages) of the most abundant clusters.

Generation times in woody bamboos are sometimes as low as several years but more commonly 30–60 years, with a 120-year cycle documented in one species of Arundinarieae (Janzen, 1976; Guerreiro, 2014). These long generation times presumably limit the informativeness of plastid or nuclear sequenced-based analyses due to slower base mutation or substitution rates (Gaut et al., 1997; Smith and Donoghue, 2008; Bromham, 2009; Ma et al., 2017), making phylogenetic estimation in this group particularly challenging (e.g., Triplett and Clark, 2010; Kelchner and Bamboo Phylogeny Group, 2013). However, even though TEs can be removed within a few million years from some genomes (Ma et al., 2004; Wicker and Keller, 2007; Devos, 2010; Legrand et al., 2019), the long generation times in woody bamboos likely enable the detection of evolutionary patterns or processes from earlier in the history of this lineage than would normally be expected based on the analysis of repeat diversity.

That 365 of 655,417 repeat clusters account for half of the total number of clustered reads indicates that a small number of repeats (in particular TE families) are responsible for most of the repeats in the genome, a result that is consistent with many angiosperm genomes over > 1 Gb (Bennetzen and Wang, 2014). The 365 most-abundant clusters are probably the most recently amplified families, as shown in other studies by their high degree of within-family identity (Park et al., 2021). Of the 365 clusters, we infer that the 86 shared by the four clades represent families that were both retained from the common ancestor of the bamboo lineage and have been recently active. The higher diversity of repeats in woody bamboos compared to the herbaceous clade may be the result of multiple hybridization, polyploidization and/or activation events (Bennetzen and Wang, 2014; Triplett et al., 2014).

The analysis of shared repeat cluster diversity (Figures 3, 4) sheds light on patterns of genome similarity and, thus, on deep relationships involved in the origins of bamboo clades. Guo et al. (2019) inferred four diploid ancestors (genomes A, B, C, and D) of the bamboos, with the C genome shared by the three extant lineages of woody bamboos. The large number of clusters shared by the Arundinarieae, the PWB and the NWB seemingly supports the idea of a shared genome, yet the NWB was found to share more repeat clusters (14) with the Olyreae than with the Arundinarieae (4) or the PWB (7). Moreover, the Olyreae shared none with the PWB and only 1 with the Arundinarieae (Figure 3).

In contrast, the main repeat cluster formed by the Olyreae and the NWB (Figure 4) suggests that the NWB share an ancestral nuclear genome with the Olyreae. This would explain the plastome shared by the Olyreae and the NWB, and the asymmetry in shared repeat clusters can be explained by biased nuclear fractionation in favor of the Olyreae genome following ancient hybridization (Wu et al., 2015; Wysocki et al., 2015, 2016). Alternatively, the transient polyploidy formed by the merging of an ancestral Olyreae and ancestral woody genome could have been followed by the preferential loss of the Olyreae chromosome segments, leading to a return to diploid status. However, any TEs active in the Olyreae ancestor could have transposed into the preferentially retained woody bamboo chromosome segments, and thus have survived the eventual elimination (or near elimination) of Olyreae genic DNA. The complete loss of one parental genome from a wide cross is well-documented for maize X oat crosses (Riera-Lizarazu et al., 1996), and is the basis of wheat haploid generation in maize X wheat crosses (Laurie and O’Donoughue, 1994) or wheat X wild barley (Inagaki and Tahir, 1990). Moreover, the transposition of TE families distinctive to one genome parent in an allopolyploid origin into the other parental genome has been documented in teff and several other genomes and, in fact, has been used to date the origin of the allopolyploidy event (Abrouk et al., 2020; VanBuren et al., 2020).

Within the NWB, the Chusqueinae cluster is distinct from the Arthrostylidiinae + Guaduinae cluster, consistent with the often less than robust support for the monophyly of the NWB (e.g., Kelchner and Bamboo Phylogeny Group, 2013; Triplett et al., 2014; Wysocki et al., 2015), different evolutionary rates between the two lineages (e.g., Kelchner and Bamboo Phylogeny Group, 2013; Wysocki et al., 2015; Wang et al., 2020), or their differing genome sizes (see below). Within Olyreae, the Parianinae and Olyrinae clusters in the Olyreae reflect the signal from both plastid and nuclear markers (e.g., Ferreira et al., 2019; Wang et al., 2020). Moreover, similarities in the repeat clusters between the Olyrinae and the Arthrostylidiinae + Guaduinae suggest shared ancestral genomes.

The PWB and the Arundinarieae formed the other main cluster in the correlation matrix (Figure 4), and this similarity as well as the large number of clusters they share (Figure 3) suggest that these two lineages share an ancestral genome. Biased fractionation is the obvious explanation for the asymmetry in shared clusters, although in this instance it favored retention of the nuclear DNA of the ancestral woody genome in the PWB. Within the PWB, a Melocanninae cluster (*Cephalostachyum* and *Schizostachyum*) is completely consistent with the available nuclear phylogenetic signal (Goh et al., 2013) and all plastid-based analyses to date (e.g., Goh et al., 2013; Zhou et al., 2017b), and supports the early divergence of this clade from the remainder of the PWB. The clustering of *Nastus elatus* and *Dinochloa* is also not surprising, as they are part of a larger clade that is consistently recovered in both plastid and nuclear analyses (Goh et al., 2013; Zhou et al., 2017b), but the lack of similarity with *Neololeba* is unexpected, since it is usually part of the same larger clade. The intermixing and strong similarity of the remaining taxa, which all belong to the *Bambusa-Dendrocalamus-Gigantochloa* (BDG) complex, is consistent with the reticulate evolution documented in this clade (Goh et al., 2013; Zhou et al., 2017b; Liu et al., 2020).

The pattern within the Arundinarieae is similar in many respects. The clustering of *Ampelocalamus* and *Drepanostachyum* reflects their phylogenetic proximity based on both plastid and nuclear data (Yang et al., 2013; Zhang et al., 2016), and the position of this clade as a relatively early-diverging lineage within the tribe based on ddRAD data (summarized in Zhang et al., 2020). This clade also exhibits a different genome size from the rest of the tribe (see below). The clustering of *Oldeania* with these two genera perhaps represents an instance of biased fractionation. The intermixing of the remaining taxa may reflect similarity due to vertical inheritance in some cases (e.g., the groupings of species of *Fargesia* or *Phyllostachys*) but homoploid hybridization is pervasive in this group (Triplett et al., 2014; Triplett and Clark, 2021) and, considering the hypothesis of rapid and relatively recent diversification (starting 7–8 mya) in the group (Zhang et al., 2016), the effects of incomplete lineage sorting cannot be ruled out.

### Genome Sizes Suggest Downsizing and Diploidization in Some Bamboo Lineages

Overall, our genome size measurements are consistent with previous analyses (Gielis et al., 1997; Kumar et al., 2011; Zhou et al., 2017a; Zappelini et al., 2020). Results are similar for most species that were already measured, with two sets of exceptions. First, *Bambusa oldhamii* was ∼4.25 pg in our analysis versus ∼2.71 pg and *B. variostriata* was ∼2.77 pg versus 4.19 pg in Zhou et al., 2017a. Both species (as *Dendrocalamopsis* in Li et al., 2001) have a range of reported chromosome numbers that are consistent with hexaploidy, which is general in *Bambusa*, *Dendrocalamus*, and *Gigantochloa*, and also with octoploidy, which is uncommon across these three genera. If the octoploid individuals or populations in these two species are of recent origin, larger genome sizes would be expected, and the discrepancy in values may simply reflect sampling. Second, Zappelini et al. (2020) reported values of 3.98 pg and 3.99 pg for *Guadua chacoensis* and *G*. *angustifolia*, respectively, whereas we measured *G*. *angustifolia* at 2.99 pg. Although different standards were used (rice in the present study and soybean in Zappelini et al., 2020), this seems unlikely to produce such a large difference. *Guadua angustifolia* is a widely cultivated species and also exhibits annual flowering without culm death (Judziewicz et al., 1999), so variation in genome size might be expected. And as noted below, the possibility of TE amplification cannot be excluded. Whatever the cause, this discrepancy merits further investigation, but does not substantially affect our main conclusions.

Although genome size variation is strongly correlated with ploidy in closely related lineages, very rapid changes in genome size also can occur without variation in ploidy. For instance, the wild maize relative, *Zea luxurians*, more than doubled its genome size without polyploidy or change in chromosome number in less than 3 million years through the action of extensive TE amplification (Estep et al., 2013). Hence, chromosome numbers are usually a better indicator of ploidy status than is genome size.

Genome sizes within the Arundinarieae and the PWB are relatively consistent, with a few exceptions. In the PWB, as noted above, the value for *B*. *oldhamii* is an outlier. The two *Neohouzeaua* samples produced very different values (2.63 and 3.25 pg). This suggests that the two samples may not represent the same introduction, although the plastome analysis supports a close relationship between them. In the Arundinarieae, *D. khasianum* and the two *Ampelocalamus scandens* accessions have much smaller genome sizes than the rest, and these values are comparable to the majority of the PWB. Based on ddRAD data, *Ampelocalamus*, *Drepanostachyum*, and *Himalayacalamus* are well supported as the second earliest-diverging lineage (the subtribe Ampelocalaminae) within the Arundinarieae (summarized in Zhang et al., 2020). The remainder of the sampled Arundinarieae, representing the two large subtribes that make up the core of this tribe, have larger genome sizes, up to approximately two times as large as those of the Ampelocalaminae or PWB. Zhou et al. (2017a) also demonstrated that, despite their smaller genomes, the PWB have a much higher number of chromosomes than the Arundinarieae.

The Olyreae show wide variation in genome sizes with large standard deviation bars for the larger genomes. The smallest genome sizes are found in two diploid species, *Raddia distichophylla* and *R. brasiliensis*, while the two largest genomes are from the polyploids *Pariana radiciflora* and *Eremitis sp*., inferred to be tetraploid or hexaploid and/or octoploid, respectively (Judziewicz et al., 1999). The large size of these two genomes coupled with polyploidy could be the result of more recent WGDs, as there is no evidence of genome downsizing or diploidization (Escudero and Wendel, 2020). Finally, within the NWB clade, the Arthrostylidiinae + Guaduinae have mostly smaller genome sizes comparable to those of the PWB, whereas the genome size range of Chusqueinae species is larger and comparable to that of the core Arundinarieae. Despite this difference, Zappelini et al. (2020) detected only one pair of rDNA and heterochromatic bands in one species of *Chusquea* and two of *Guadua*, suggesting diploidization in both lineages of the NWB. Taken together, the genome size data and cytogenetic evidence strongly suggest that genome downsizing and diploidization have taken place in the PWB, the Arthrostylidiinae + Guaduinae clade of the NWB, and the Ampelocalaminae of the Arundinarieae. The situation with respect to the Chusqueinae and the core Arundinarieae is less clear, but either genome downsizing did not occur or genome fluctuations did not change overall genome size prior to diversification in these lineages.

### The Evolutionary History of Woody Bamboos: Integrating Ancient Hybridization, Allopolyploidy, Biased Fractionation and Diploidization

That ancient hybridization and polyploidization were involved in the origins of the three major lineages of woody bamboos is well established (Triplett et al., 2014; Guo et al., 2019). However, evidence from our analysis of repeat diversity also supports biased fractionation as an important factor in the evolution of the woody bamboos, and our genome size data support instances of trends toward diploidization in all three lineages. Not excluding the possibility that repeats may have independently and differentially amplified in the extant lineages, we here provide a novel and synthetic perspective on the evolution of bamboos, including a scenario to explain the origins of the three woody bamboo lineages while resolving the apparent conflict in the plastid vs. nuclear signals.

**FIGURE 6:**
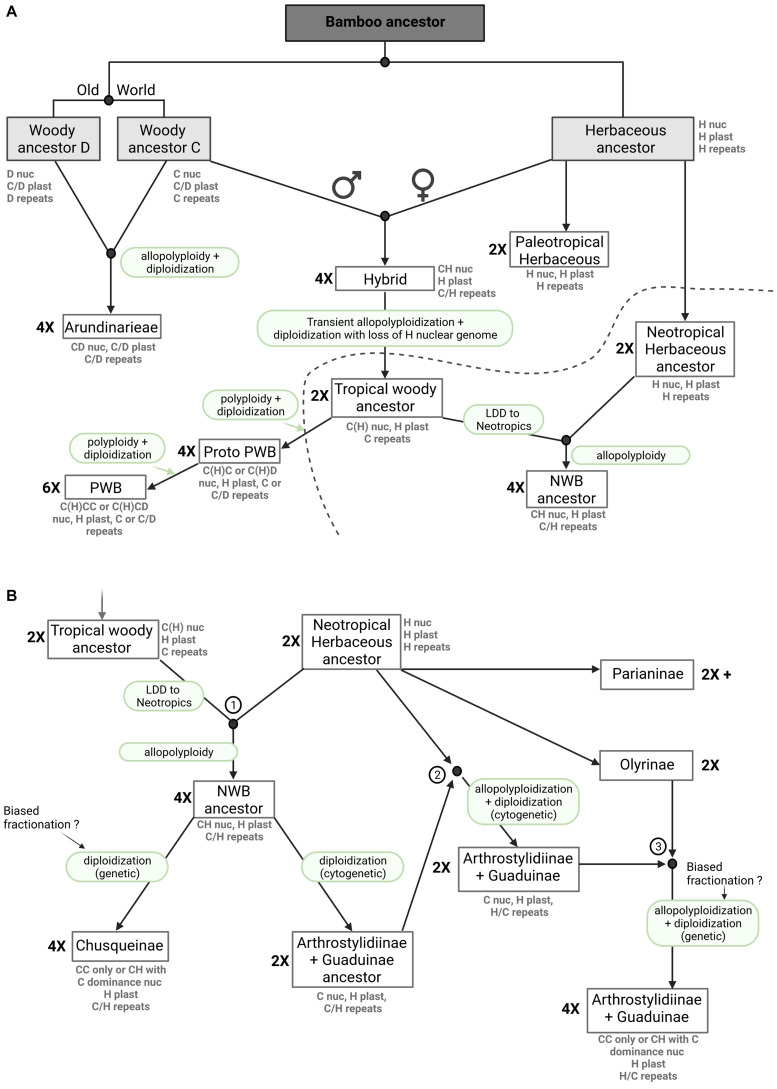
Bamboo origins and evolution, a novel hypothesis. The presented figure depicts a model of bamboo origins and evolution that includes ancient hybridization, allopolyploidy, biased fractionation, genome dominance and diploidization. Nuclear and plastome genomes, as well as repeats (mobilomes, including TEs and other repeats) are presented for each extant clade. The ploidy level (2X, 4X, 6X) is indicated on the left side of the boxes. **(A)** Evolutionary hypothesis from the common bamboo ancestor to extant Arundinarieae, extant Paleotropical Woody Bamboos (PWB) and the Neotropical Woody Bamboo (NWB) ancestors. **(B)** Evolutionary hypothesis leading to the different extant NWB lineages after allopolyploidization between tropical woody and Neotropical herbaceous ancestors. The three encircled numbers match the three hybridization events denoted in Figure 4. LDD means long distance dispersal.

The origin and evolution of the Arundinarieae is relatively straightforward. All evidence to date supports ancient hybridization between two diploid genomes derived from a common ancestor with subsequent allopolyploidy (Triplett et al., 2014; Guo et al., 2019). The diploidization inferred for the Ampelocalaminae almost certainly involved biased fractionation, but if the two ancestral genomes were similar enough, this would not necessarily be detectable in the repeat analyses and a shared plastome would be expected in any case (Figure 6A). If the core lineage of Arundinarieae, which shows no evidence of diploidization, underwent rapid and relatively recent diversification (Peng et al., 2013; Zhang et al., 2016), this occurred after the divergence of the Ampelocalaminae.

The plastid signal unambiguously demonstrates that the Olyreae and the Bambuseae share a plastome (Kelchner and Bamboo Phylogeny Group, 2013; Wysocki et al., 2015). Under the scenarios proposed by Triplett et al. (2014) or Guo et al. (2019), the only possible explanation would be ancestral polymorphism in plastomes retained for up to 9 my before the divergence of the diploid ancestors of the tropical (Bambuseae) and temperate (Arundinarieae) woody bamboo lineages. And if correct, little to no similarity between the extant Olyreae nuclear genome and any of the four (or five) inferred ancestral diploid genomes of woody bamboos would be expected.

However, the analyses of repeat diversity strongly suggest that the NWB and the Olyreae recently shared the common repeat content of a nuclear genome, apparently conflicting with the genomic level signal (Guo et al., 2019). We postulate an ancient hybridization event between a maternal Olyreae genome (H) and a paternal C genome (or an ancestral woody bamboo genome) with transient allopolyploidy, followed by the preferential loss of most of the Olyreae nuclear chromosome segments, leading to a return to diploid status in this tropical woody ancestor, with the potential survival of some TEs from the Olyreae ancestor (Figure 6A). This scenario would explain the plastome shared by the Olyreae and both the NWB and PWB (Wu et al., 2015; Wysocki et al., 2015, 2016), the asymmetry in shared repeat clusters between the Olyreae and the NWB but also the presence of a few repeat clusters shared between Olyreae and the PWB, and the coding nuclear DNA signal shared by the NWB with the other woody bamboo lineages (Triplett et al., 2014; Guo et al., 2019). Thus, it is possible to propose one ancient hybridization event involving the C and H genomes, with an early split from a tropical woody ancestor leading to the NWB and PWB lineages, but in this case a long distance dispersal event of the tropical woody ancestor prior to another hybridization with the herbaceous ancestor must be inferred (Figure 6A). Under an alternative scenario, both biogeography and the mechanics of allopolyploid formation would favor separate and not necessarily contemporaneous hybridization events leading to the NWB and PWB lineages. The effective population size of a recently formed allopolyploid is likely to be small and unlikely to exhibit polymorphism (Wendel et al., 2018). The NWB are entirely Neotropical and the PWB are entirely Paleotropical, whereas in the Olyreae, the Parianinae and Olyrinae are Neotropical (the one species also found in Africa is a recent arrival, Ruiz-Sanchez et al., 2019) while their sister lineage is Paleotropical (Kelchner and Bamboo Phylogeny Group, 2013; Ruiz-Sanchez et al., 2019; Figures 6A,B), making it possible for an ancestral H genome to combine with a C genome in Asia or the Americas. For the sake of simplicity, we show the first scenario in Figure 6A.

In our model, we propose a hybridization event between the diploid tropical woody ancestor and the diploid Neotropical herbaceous ancestor leading to the tetraploid NWB ancestor harboring both C and H genomes and repeats (Figure 6B, 1). Diploidization, with complete or near-complete loss of H chromosome genes leads to two lineages, the extant Chusqueinae and an Arthrostylidiinae + Guaduinae ancestor. With respect to the intermediate repeat cluster observed in Figure 4, we propose two more rounds of hybridization between the Arthrostylidiinae + Guaduinae ancestor and the Neotropical herbaceous ancestor first (Figure 6B, 2) and then the Olyrinae (Figure 6B, 3), leading to the extant tetraploid Arthrostylidiinae + Guaduinae clade harboring a dominant woody C genome with some H signatures and both H and C repeats. Extant members of both Olyreae and the Arthrostylidiinae + Guaduinae clade are often sympatric and tend to grow in the same, relatively low elevation habitats, and assuming their ancestors were similar, opportunities for hybridization would have been available (Judziewicz et al., 1999; Ruiz-Sanchez et al., 2021).

The PWB share a nuclear genome with the Arundinarieae, as inferred from the repeat analyses, yet share a plastome with the Olyreae and the NWB. As opposed to the NWB, we propose that the PWB underwent a more straightforward evolutionary pathway than the NWB, involving two different rounds of polyploidization with biased fractionation in favor of the C genome and giving rise to the extant PWB clade (Figure 6A).

Previous nuclear analyses failed to explain the plastome shared by Bambuseae and Olyreae. Although Triplett et al. (2014) sampled all major bamboo lineages, only three nuclear markers were used. By chance, it is possible that all three reflected the shared woody bamboo genome (Guo et al., 2019), leading to the inference of woody bamboo monophyly with no nuclear contribution from the Olyreae. On the other hand, Guo et al. (2019) included the largest amount of genomic data with all clades represented in a bamboo study to date, but they sampled only one species per woody clade and two species of Olyreae, and used only one of the Olyreae species in their genomic analysis. They analyzed syntenic blocks of genes conserved between bamboo species in order to investigate the evolution of the woody syndrome. However, as predicted by our scenario, if the coding nuclear DNA of the Olyreae was preferentially lost in each of the NWB and PWB lineages due to biased fractionation, the woody bamboos would indeed be inferred as monophyletic based on nuclear DNA. Interestingly, Guo et al. (2019) mentioned that not all topologies supported the monophyly of the woody bamboos. In order to extend previous analyses, we performed phylogenetic reconstructions independently on 20 genes using six available full genome sequences (Supplementary Material and Supplementary Figure 3). In 10% of the cases, the topology does not show the monophyly of the woody bamboos, supporting our scenario, especially the retention of some Olyreae genic regions in the PWB.

## Conclusion and Perspectives

We successfully combined information from plastome phylogenetic reconstruction, repeat analyses and genome size estimations of a large number of species representing all major lineages to detect ancient hybridization, diploidization and genome fractionation signals in the evolutionary history of bamboos. Our hypothesis confirms ancient hybridization and polyploidy in the origins of the extant woody bamboo lineages, as postulated previously, but requires only three ancestral diploid genomes (versus four or five), of which one (H) was herbaceous, and explains the plastome shared by the Olyreae and Bambuseae. The C genome, shared by all three woody lineages, must have evolved the woody syndrome prior to the ancient hybridization events. Evolution of the woody syndrome prior to the divergence of the C and D genomes may be even more likely, given their apparent similarity. Biased fractionation and diploidization explain the similarity in the repeat signal between the Arundinarieae and the PWB and between the NWB and the Olyreae, respectively, although the sequence of events and processes differed between the PWB and the NWB.

Further genomic-level studies will be needed to challenge our hypothesis and test the alternative possibility of differential repeat amplification between lineages, and to obtain a better understanding of the evolutionary history of bamboos, especially with respect to the process of genome fractionation, including subgenome dominance and genomic rearrangements. Polyploidization events combined with a slow evolutionary rate in the woody bamboos make the Bambusoideae a fascinating lineage in which to study diploidization and associated processes. A separate analysis of diploid and polyploid species would give more insights into these processes. Unfortunately our sampling only includes two diploid species, making this type of analysis impossible to perform. We focused our analyses on plastome reconstruction and repeats as both are easily found in low-coverage SSA. This low-coverage data does not allow us to fully reconstruct bamboo transcriptomes. The signal from gene-based reconstruction would be too weak to establish which of the parental genomes dominate. Higher coverage sequencing and whole genome assembly will be needed to enable comparative genomics, including chromosome ancestral reconstruction, to investigate and quantify gene retention or loss, and to assign genes to the parental genomes as additional possible ways to test our scenario (Murat et al., 2014; Renny-Byfield et al., 2015; Emery et al., 2018).

## Data Availability Statement

The datasets presented in this study can be found in online repositories. The names of the repository/repositories and accession number(s) can be found below: https://www.ebi.ac.uk/ena, PRJEB43575.

## Author Contributions

DC, LC, and JB designed the study. DC and MP performed the transposable elements and phylogenetic analyses. WW and MD performed the plastome assemblies. DC, LC, and JB wrote the manuscript. All the authors contributed to the article and approved the submitted version.

## Conflict of Interest

The authors declare that the research was conducted in the absence of any commercial or financial relationships that could be construed as a potential conflict of interest.

## Publisher’s Note

All claims expressed in this article are solely those of the authors and do not necessarily represent those of their affiliated organizations, or those of the publisher, the editors and the reviewers. Any product that may be evaluated in this article, or claim that may be made by its manufacturer, is not guaranteed or endorsed by the publisher.

## Footnotes

1
http://dna.uga.edu

2
http://mrbayes.sourceforge.net

3
http://www.bioinformatics.babraham.ac.uk/projects/trim_galore/

## Supplementary Material

Supplementary Figure 1 Phylogenetic reconstruction with 115 bamboo plastomes. The phylogenetic reconstruction is based on the alignment of 115 full plastome sequences. Within the 115 sequences, 51 correspond to the species used in the main text and sequenced for this study (green font), 50 correspond to previously published plastomes (available on database) and two are non-bamboo species used as outgroups (*Lolium* and *Zizania aquatica*). Statistical support is shown at the nodes.

Supplementary Figure 2 Maximum Likelihood phylogenetic reconstruction with 51 species.

Supplementary Figure 3 Phylogenetic topologies of 20 nuclear genes. Branches of the Olyreae clade are green-colored.

Supplementary Table 1 Additional plastomes included from GenBank for the 115-plastome analysis.

Supplementary Table 2 Genome size values of the 51 sequenced bamboos.
