# Supplementary material for: Integrated Genomic Analyses From Low-Depth Sequencing Help Resolve Phylogenetic Incongruence in the Bamboos (Poaceae: Bambusoideae)
Source: Front Plant Sci. 2021 Sep 3;12:725728. doi: 10.3389/fpls.2021.725728 (PMC8456298; doi:10.3389/fpls.2021.725728)
Supplement: Supplementary Figure 1 — Phylogenetic reconstruction with 115 bamboo plastomes. The phylogenetic reconstruction is based on the alignment of 115 full plastome sequences. Within the 115 sequences, 51 correspond to the species used in the main text and sequenced for this study (green font), 50 correspond to previously published plastomes (available on database) and two are non-bamboo species used as outgroups (Lolium and Zizania aquatica). Statistical support is shown at the nodes. [file Data_Sheet_1.docx]

Supplementary Material

# Supplementary Information

## Gene-based phylogenies

Available published genomes (cDNA libraries) of *Phyllostachys heterocycla* (named TWB_Phet, Peng et al., 2013; http://server.ncgr.ac.cn/bamboo/), *Phyllostachys edulis* (named TWB_Pedu, Zhao et al., 2014; http://www.bamboogdb.org/#/), *Raddia guianensis* (Oly_Rdui, Guo et al., 2019), *Olyra latifolia* (Oly_Olat, Guo et al., 2019), *Guadua angustifolia* (NWB_Gang, Guo et al., 2019) and *Bonia amplexicaulis* (PWB_Bamp, Guo et al., 2019) were downloaded to infer a nuclear phylogenetic topology of 20 randomly selected nuclear genes. Annotated cDNA of *P. heterocycla* were used as query sequences to perform blastn analysis in the other five cDNA libraries. Sequences were then aligned in Seaview (Gouy et al., 2010) using Muscle aligner and PhyML with optimized parameters.

Phylogenetic reconstructions were performed for the following genes: ataxin-2 related protein (ID=PH01000133G0570); 3-ketoacyl-CoA synthase (ID=PH01002162G0290); DTA2 (ID=PH01000001G1880); FERONIA receptor-like kinase (ID=PH01000000G0250), GAPDH (NCBI ID=KF484750.1 from *Dendrocalamus latiflorus*); glyoxalase family protein (ID=PH01001542G0400); GRAS family transcription factor domain containing protein (ID=PH01000000G1660); KIP1 (ID=PH01000133G1440); methyltransferase (ID=PH01000000G3540); Nitrilase (ID=PH01000006G0430); polyadenylate-binding protein (ID=PH01000056G1770); PRR95 (NCBI ID=NM_001357737.1 from maize); peptide transporter PTR2 (ID=PH01000187G1190); Regulator of chromosome condensation domain containing protein (RCC1, ID=PH01005728G0040); retinoblastoma-related protein-like (ID=PH01002621G0030); SCC3 (ID=PH01005830G0010); SKP1 (ID=PH01000287G0520); Endoglucanase (PH01001590G0100); SNF2 family N-terminal domain containing protein (ID=PH01000000G4660); VTC2 (ID=PH01000031G0320).

# Supplementary references

Gouy, M., Guindon, S., and Gascuel, O. (2010). SeaView version 4 : a multiplatform graphical user interface for sequence alignment and phylogenetic tree building. *Mol. Biol. Evol.* 27, 221-224. doi: 10.1093/molbev/msp259.

Zhao, H., Peng, Z., Fei, B., Li, L., Hu, T., Gao, Z., et al. (2014). BambooGDB: a bamboo genome database with functional annotation and an analysis platform. *Database* 2014:bau006. doi: 10.1093/database/bau006.

# Supplementary Figures and Tables


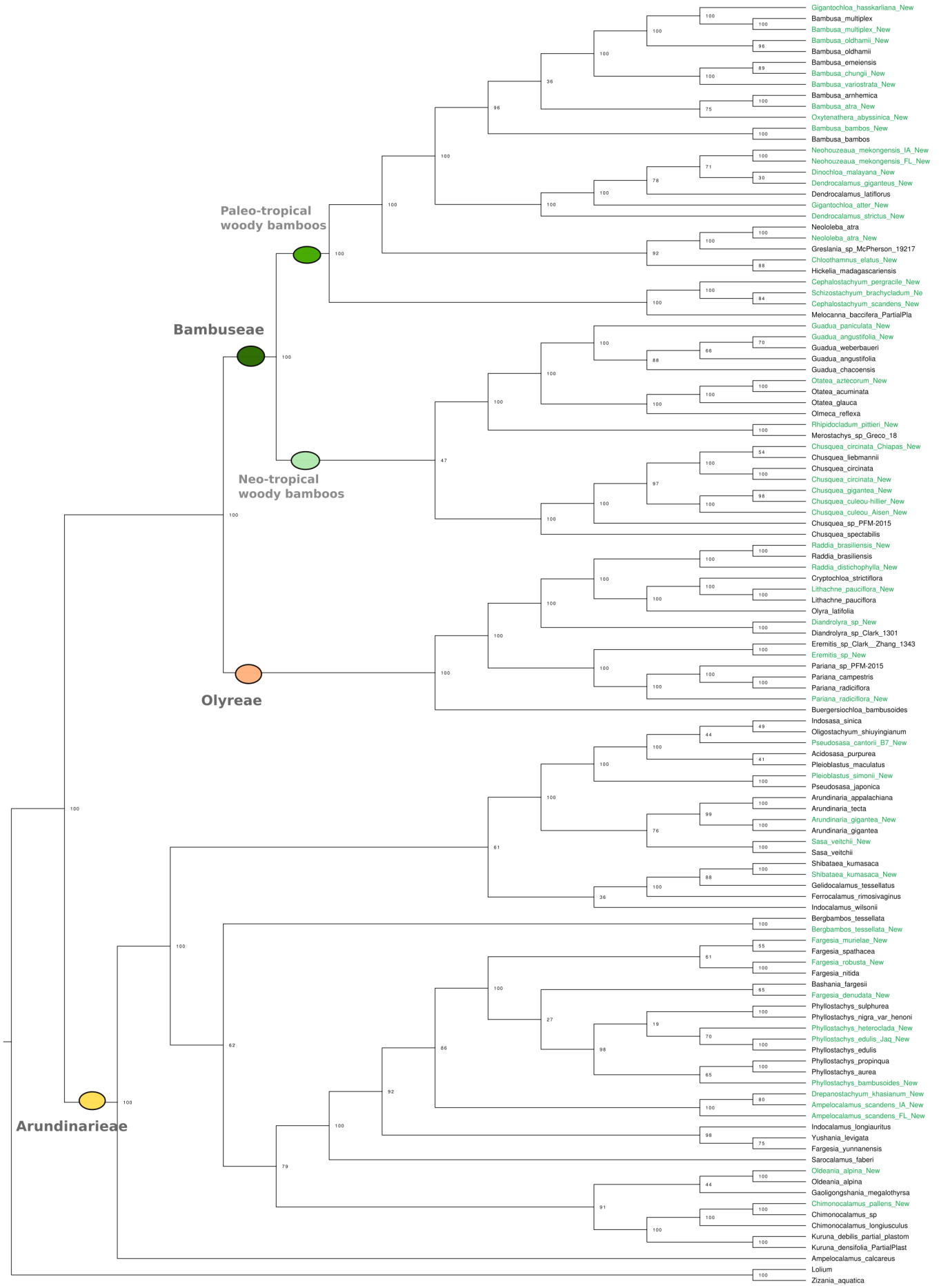


**Supplementary Figure 1.** Phylogenetic reconstruction with 115 bamboo plastomes. The phylogenetic reconstruction is based on the alignment of 115 full plastome sequences. Within the 115 sequences, 51 correspond to the species used in the main text and sequenced for this study (green font), 50 correspond to previously published plastomes (available on database) and two are non-bamboo species used as outgroups (*Lilium* and *Zizania aquatica*). Statistical support is shown at the nodes.


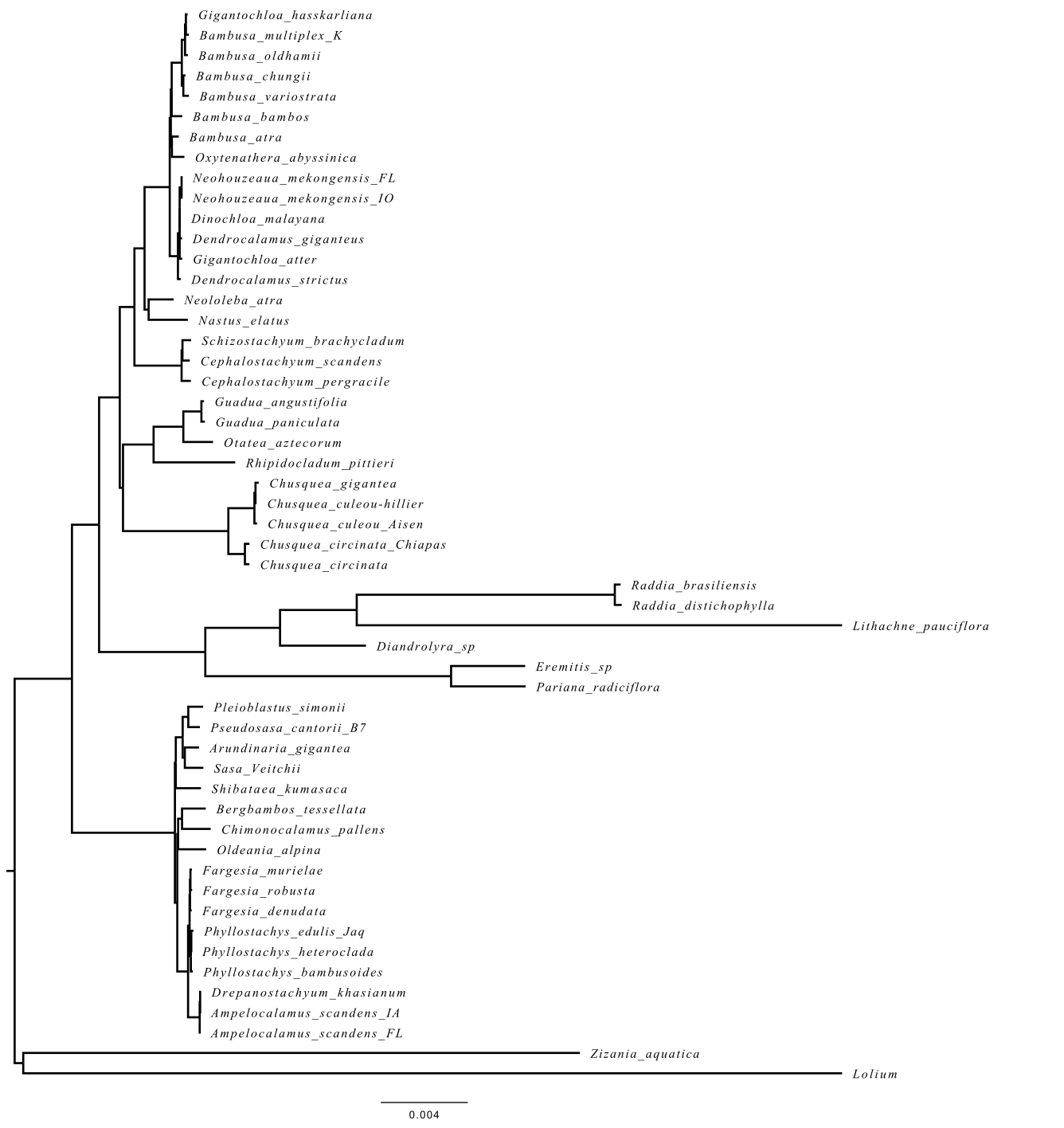


**Supplementary Figure 2.** Maximum Likelihood phylogenetic reconstruction with 51 species.


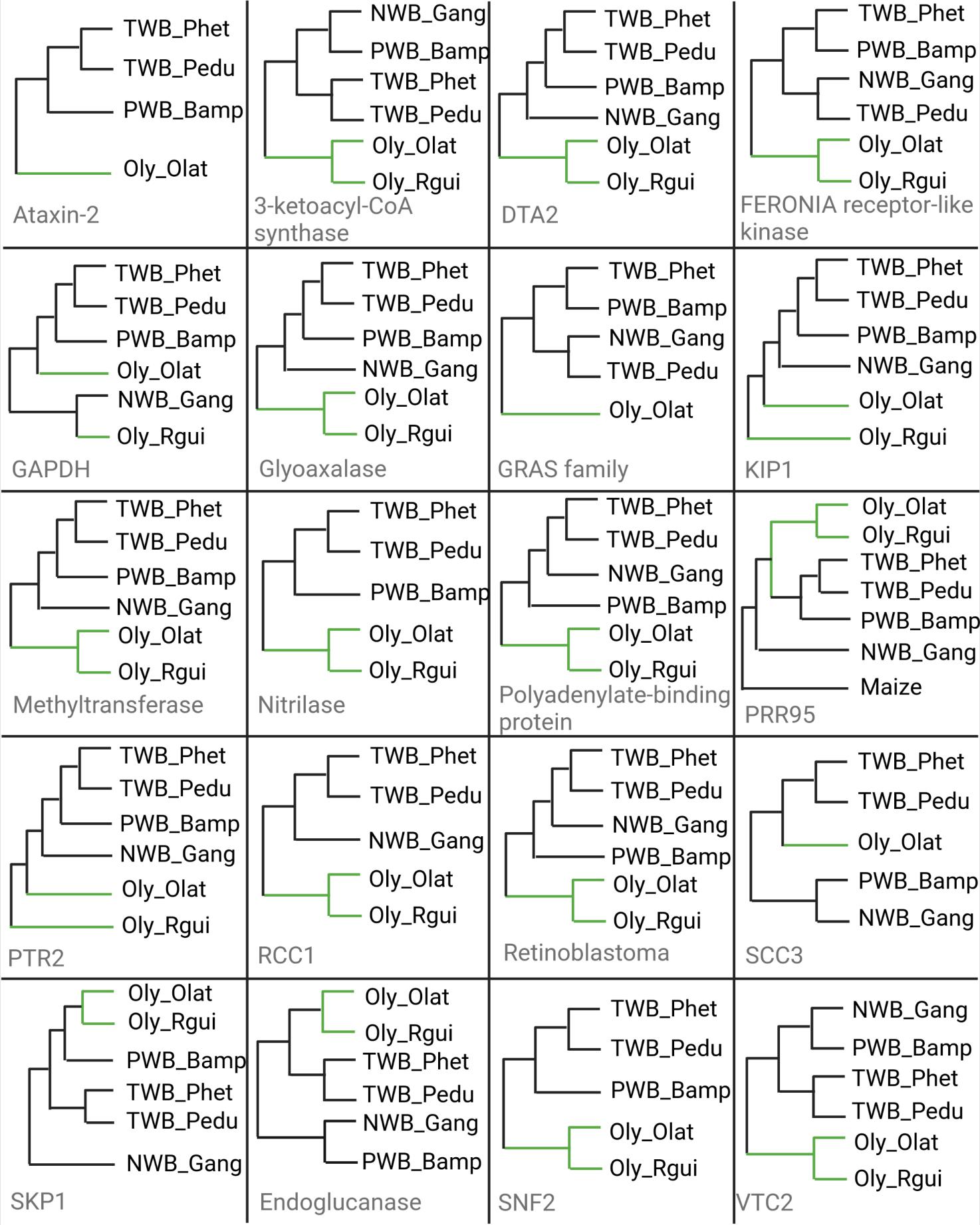


**Supplementary Figure 3.** Phylogenetic topologies of 20 nuclear genes. Branches of the Olyreae clade are green-colored.

**Supplementary Table 1.** Additional plastomes included from GenBank for the 115-plastome analysis.

| **Taxon** | **Voucher or Living Collection & Plot Number** | **GenBank Number** |
| --- | --- | --- |
| **OLYREAE** |  |  |
| *Buergersiochloa bambusoides* | Dransfield 1365 (K) | KJ871000 |
| *Cryptochloa strictiflora* | Davidse 35629 (MO) | JX235348 |
| *Diandrolyra sp.* | Clark 1301 (ISC) | KJ870991 |
| *Eremitis sp.* | Clark & Zhang 1343 (ISC) | KJ870992 |
| *Lithachne pauciflora* | Clark 1297 (ISC) | KJ871002 |
| *Olyra latifolia* | AF 97 | KF515509 |
| *Pariana campestris* | ??? | KP319244 |
| *Pariana radiciflora* | Clark & Zhang 1344 (ISC) | KJ871004 |
| *Raddia brasiliensis* | Clark & Attigala 1713 (ISC) | KJ870998 |
| **ARUNDINARIEAE** |  |  |
| *Acidosasa purpurea* | Zhang 08023 (KUN) | HQ337793 |
| *Ampelocalamus calcareus* | MPF10050 (KUN?) | KJ496369 |
| *Ampelocalamus naibunensis* | Zhang12318 (KUN) | KX372537 |
| *Arundinaria appalachiana* | Triplett 099 (MO) | KC817462 |
| *Arundinaria gigantea* | Burke s.n. (DEK) | JX235347 |
| *Arundinaria tecta* | Triplett 173 (MO) | KC817463 |
| *Bashania (Arundinaria) fargesii* | MPF10139 (KUN?) | JX513413 |
| *Bergbambos tessellata* | Clark 1318 (ISC) | KU569967 |
| *Bergbambos tessellata* | KMBG1301 (KUN) | KJ522748 |
| *Chimonocalamus longiusculus* | MPF10182 (KUN?) | JX513415 |
| *Chimonocalamus sp.* | Clark & Reiners s.n. (ISC) | KU523577 |
| *Fargesia nitida* | Saarela 597531 | KU569968 |
| *Fargesia spathacea* | MPF10141 (KUN?) | JX513417 |
| *Fargesia yunnanensis* | MPF10162 (KUN?) | JX513418 |
| *Ferrocalamus rimosivaginus* | Zhang 08019 (KUN) | HQ337794 |
| *Gaoligongshania megalothyrsa* | MPF10056 (KUN?) | JX513419 |
| *Gelidocalamus tessellatus* | MPF10049 (KUN?) | JX513420 |
| *Indocalamus longiauritus* | MPF 10168 (KUN) | HQ337795 |
| *Indocalamus wilsonii* | MPF10146 (KUN?) | JX513421 |
| *Indosasa sinica* | ??? | JX513422 |
| *Kuruna debilis (partial)* | Attigala 123-5 (ISC) | KU569969 |
| *Oldeania alpina* | Attigala 170 (ISC) | KU569972 |
| *Oligostachyum shiuyingianum* | ??? | JX513423 |
| *Phyllostachys aurea* | Attigala 172 (ISC) | KU569973 |
| *Phyllostachys edulis* | MPF 10163 (KUN) | HQ337796 |
| *Phyllostachys nigra var. henonis* | MPF 10172 (KUN) | HQ154129 |
| *Phyllostachys propinqua* | No voucher | JN415113 |
| *Phyllostachys sulphurea* | ??? | KJ722540 |
| *Pleioblastus maculatus* | MPF10161 (KUN?) | JX513424 |
| *Pseudosasa hindsii (partial)* | Clark 1317 (ISC) | KU569974 |
| *Pseudosasa japonica* | ???; unpublished | KT428377 |
| *Sarocalamus faberi* | CZM025 (KUN?) | JX513414 |
| *Sasa veitchii* | Clark 1325 (ISC) | KU569975 |
| *Shibataea kumasaca* | Clark 1290 (ISC) | KU523578 |
| *Thamnocalamus spathiflorus* | Clark 1319 (ISC) | KJ871005 |
| *Yushania levigata* | YD02 (KUN?) | JX513426 |
| **BAMBUSEAE** |  |  |
| *Bambusa arnhemica* | CAN: 1846 | KJ870989 |
| *Bambusa bambos* | Bogor Botanical Garden BI-1 | KJ870988 |
| *Bambusa emeiensis* | MPF10170 (KUN) | HQ337797 |
| *Bambusa multiplex* | ??? | KJ722536 |
| *Bambusa oldhamii* | ??? | FJ970915 |
| *Chusquea circinata* | ??? | KP319241 |
| *Chusquea liebmannii* | Clark & Attigala 1710 (ISC) | KJ871001 |
| *Chusquea spectabilis* | Londoño & Clark 919 (ISC) | KJ870990 |
| *Dendrocalamus latiflorus* | ??? | FJ970916 |
| *Greslania sp.* | McPherson 19217 (MO) | NC_026961 |
| *Guadua angustifolia* | Hua’an Bamboo Garden, Fujian Province,  China | KM365071 |
| *Guadua chacoensis* | Greco 159 (FLOR) | KT373814 |
| *Guadua weberbaueri* | Londoño & Kobayashi 582 (TULV) | KP793062 |
| *Hickelia madagascariensis* | S. Dransfield 1349 (K) | KJ870994 |
| *Melocanna baccifera (partial)* | Londoño & Clark 930 | KU569971 |
| *Merostachys sp.* | Greco 18 (FLOR) | KT373815 |
| *Neohouzeaua sp.* | Clark & Attigala 1712 | KJ870995 |
| *Neololeba atra* | Clark & Triplett 1663 | KJ870996 |
| *Olmeca reflexa* | Francisco Botanical Garden 312 | KJ870997 |
| *Otatea acuminata* | Clark & Zhang 1348 (ISC) | KJ871003 |
| *Otatea glauca* | ??? | KP319243 |

**Supplementary Table 2.** Genome size values of the 51 sequenced bamboos.

| Species abbreviation | Species | Clade | GS2C (pg/2C) | StDev | GSMb (Mb) |
| --- | --- | --- | --- | --- | --- |
| Rdis | *Raddia distichophylla* | Herbaceous | 1.37 | 0.033 | 669.9 |
| Rbra | *Raddia brasiliensis* | Herbaceous | 1.91 | 0.015 | 933.99 |
| Lpau | *Lithachne pauciflora* | Herbaceous | 2.875 | 0.088 | 1405.9 |
| Dspe | *Diandrolyra* sp. | Herbaceous | 3.46 | 0.226 | 1691.9 |
| Prad | *Pariana radiciflora* | Herbaceous | 5.845 | 0.35 | 2858.2 |
| Espe | *Eremitis sp.* | Herbaceous | 8.32 | 0.403 | 4068.5 |
| Gpan | *Guadua paniculata* | NWB | 2.04 | 0.04 | 997.56 |
| Oazt | *Otatea acuminata aztecorum* | NWB | 2.41 | 0.042 | 1178.5 |
| Gang | *Guadua angustifolia* | NWB | 2.99 | 0.073 | 1462.11 |
| Rpit | *Rhipidocladum pittieri* | NWB | 3.29 | 0.046 | 1608.8 |
| Cgig | *Chusquea gigantea* | NWB | 4.05 | 0.044 | 1980.45 |
| Ccuh | *Chusquea culeou ‘*Hillier’ | NWB | 4.11 | 0.05 | 2009.79 |
| CcuA | *Chusquea culeou* Aisen II | NWB | 4.26 | 0.063 | 2083.14 |
| CciC | *Chusquea circinata* ‘Chiapas’ | NWB | 4.56 | 0.071 | 2229.84 |
| Ccir | *Chusquea circinata* | NWB | 4.58 | 0.057 | 2239.62 |
| NmeI | *Neohouzeaua mekongensis* IA | PWB | 2.63 | 0.048 | 1286.1 |
| Oaby | *Oxytenanthera abyssinica* | PWB | 2.69 | 0.033 | 1315.4 |
| Bbam | *Bambusa bambos* | PWB | 2.74 | 0.068 | 1339.86 |
| Bmuk | *Bambusa multiplex* ‘Alphonse Karr’ | PWB | 2.75 | 0.064 | 1344.75 |
| Bvar | *Bambusa variostriata* | PWB | 2.77 | 0.089 | 1354.53 |
| Bchu | *Bambusa chungii* | PWB | 2.83 | 0.084 | 1383.87 |
| Batr | *Bambusa atra* | PWB | 2.85 | 0.065 | 1393.65 |
| Csca | *Cephalostachyum scandens* | PWB | 2.87 | 0.048 | 1403.43 |
| Cper | *Cephalostachyum pergracile* | PWB | 2.9 | 0.031 | 1418.1 |
| Natr | *Neololeba atra* | PWB | 3.05 | 0.177 | 1491.45 |
| Sbra | *Schizostachyum brachycladum* | PWB | 3.05 | 0.052 | 1491.45 |
| Dgig | *Dendrocalamus giganteus* | PWB | 3.1 | 0.05 | 1486.56 |
| Dmal | *Dinochloa malayana* | PWB | 3.1 | 0.058 | 1515.9 |
| Dstr | *Dendrocalamus strictus* | PWB | 3.18 | 0.033 | 1555.02 |
| Ghas | *Gigantochloa hasskarliana* | PWB | 3.22 | 0.071 | 1574.58 |
| NmeF | *Neohouzeaua mekongensis* FL | PWB | 3.25 | 0.071 | 1589.25 |
| Cela | *Chloothamnus (Nastus) elatus* | PWB | 3.36 | 0.057 | 1643.04 |
| Gatt | *Gigantochloa atter* | PWB | 3.47 | 0.046 | 1696.83 |
| Bold | *Bambusa oldhamii* | PWB | 4.25 | 0.066 | 2078.25 |
| AscF | *Ampelocalamus scandens* FL | TWB | 2.55 | 0.112 | 1246.9 |
| AscI | *Ampelocalamus scandens* IA | TWB | 2.65 | 0.077 | 1295.85 |
| Dkha | *Drepanostachyum khasianum* | TWB | 2.65 | 0.068 | 1295.85 |
| Oalp | *Oldeania alpina* | TWB | 3.6 | 0.041 | 1760.4 |
| Pbam | *Phyllostachys bambusoides* | TWB | 3.9 | 0.066 | 1907.1 |
| Fmur | *Fargesia murielae* | TWB | 4.24 | 0.077 | 2073.36 |
| PedJ | *Phyllostachys edulis ‘*Jaquith’ | TWB | 4.29 | 0.064 | 2097.81 |
| Phet | *Phyllostachys heteroclada* | TWB | 4.54 | 0.05 | 2220.06 |
| Cpal | *Chimonocalamus pallens* | TWB | 4.74 | 0.074 | 2317.86 |
| Fden | *Fargesia denudata* | TWB | 4.78 | 0.057 | 2337.42 |
| Psim | *Pleioblastus simonii* | TWB | 4.83 | 0.101 | 2361.87 |
| Skum | *Shibataea kumasaca* | TWB | 4.86 | 0.036 | 2376.54 |
| Btes | *Bergbambos tessellata* | TWB | 4.88 | 0.069 | 2386.32 |
| PcaB | *Pseudosasa cantorii* Plot B-7 | TWB | 4.95 | 0.142 | 2420.55 |
| FroC | *Fargesia robusta* ‘Campbell’ | TWB | 5.14 | 0.064 | 2513.46 |
| Svei | *Sasa veitchii* | TWB | 5.53 | 0.111 | 2704.17 |
| Agig | *Arundinaria gigantea* ‘Macon’ | TWB | 6.32 | 0.046 | 3090.48 |
